# An overview of the key principles and guidelines in the management of pelvic fractures

**DOI:** 10.1177/1750458920947358

**Published:** 2020-09-08

**Authors:** Ahmed M H A M Mostafa, Harry Kyriacou, Mukai Chimutengwende-Gordon, Wasim S Khan

**Affiliations:** 1 University of Cambridge School of Clinical Medicine, Cambridge, UK; 2 Department of Orthopaedic Surgery, Addenbrooke's Hospital, Cambridge, UK

**Keywords:** Pelvic fracture / Perioperative / Classification / Complications / Orthopaedics

## Abstract

Pelvic fractures are complex injuries with a range of different presentations depending on the mechanism of trauma. Due to the morbidity and mortality of pelvic fractures, patients require thorough investigation and timely management with multidisciplinary input. Various surgical and non-surgical techniques can be used to treat pelvic fractures, as well as any associated visceral injuries. Following repair, it is important to remain vigilant for postoperative complications such as infection, sexual and urinary dysfunction, chronic pain and adverse psychological health. This article summarises the relevant UK guidance and literature and presents them in a format that follows the patient’s journey. In doing so, it highlights the key perioperative factors that need to be considered in cases of pelvic fracture.

**Provenance and Peer review:** Unsolicited contribution; Peer reviewed; Accepted for publication 10 July 2020.

## Introduction

Pelvic fractures are present in 7% of trauma patients, as reported by a recent study in the United Kingdom (UK) (Leach et al 2019). In a cohort of young adults with a median age of 39 years, high-energy road traffic accidents (RTA) were the predominant mechanism of injury, accounting for 62.9% of pelvic fractures (Giannoudis et al 2007). In elderly patients over 65 years of age, however, low-energy falls from standing height become the major cause, accounting for 83% of cases (Morris et al 2000). While studies with a young cohort report a male predominance, elderly pelvic fractures tend to occur more commonly in females (Giannoudis et al 2007, Morris et al 2000).

Pelvic fractures are commonly associated with other injuries, especially in high-energy trauma (Giannoudis et al 2007). These include serious to unsurvivable chest injuries (21.2%) and head injuries (16.9%), liver or spleen injuries (8.0%), two or more long bone fractures (7.8%), and urogenital injuries (3.7%) (Giannoudis et al 2007). According to a study of pelvic fracture patients from the United States, abdominal and pelvic haemorrhage are the main causes of death in the first 6 hours, followed by head injury from 6 to 24 hours, and multiple organ dysfunction syndrome beyond 24 hours (Vaidya et al 2016). As many deaths in pelvic fracture patients are attributable to associated injuries, early identification and treatment are crucial for survival.

The mortality rate of pelvic fracture patients in the UK has been reported as 7.3% during hospital admission, and 14.2% at three months after injury (Giannoudis et al 2007, Leach et al 2019). Polytrauma is associated with a poor prognosis and interestingly, a study looking exclusively at bleeding pelvic fracture patients with severe associated injuries in Japan has reported a staggering mortality rate of 46% (Kido et al 2008). Independent predictors of mortality in pelvic fracture patients include age, physiologic derangement, and associated head, chest and abdominal injuries (Giannoudis et al 2007). In those that survive, pelvic fractures have significant morbidity and are associated with a long-term decrease in physical functioning, problems with activities of daily living and adverse psychological health (Banierink et al 2019).

Risk factors for pelvic fractures in RTAs include no airbag deployment, a smaller vehicle size and a lateral deformation location (Stein et al 2006). Meanwhile, increasing age, cigarette smoking, a low bone mass and a tendency to fall are all risk factors for pelvic fractures in elderly individuals (Kelsey et al 2005). The overall incidence of pelvic fractures is increasing in many developed countries, alongside ageing populations (Buller et al 2016, Kannus et al 2015). In Finland, for example, there has been a 398% increase in the yearly incidence of osteoporotic pelvic fractures in elderly patients from 1970 to 2013 (Kannus et al 2015). Since pelvic fracture patients in the UK have more ICU admissions and significantly longer hospital stays than any other major trauma patients (15 vs. 8 days), a rising incidence is likely to exert a significant economic burden and strain on the healthcare system (Giannoudis et al 2007).

The National Institute for Health and Care Excellence (NICE) has produced detailed guidelines on the management of pelvic fractures (NICE 2016a, 2016b). These are complemented by guidelines from the British Orthopaedic Association (BOA) on pelvic fractures and associated urological complications (BOA 2016, 2018). This article is unique in that it combines and summarises these guidelines, as well as the relevant literature, and presents them in a format that follows the patient’s journey. By highlighting the key preoperative, intraoperative and postoperative factors in cases of pelvic fracture, this article aims to optimise the care that these patients receive.

## Clinical anatomy

**Figure 1:**
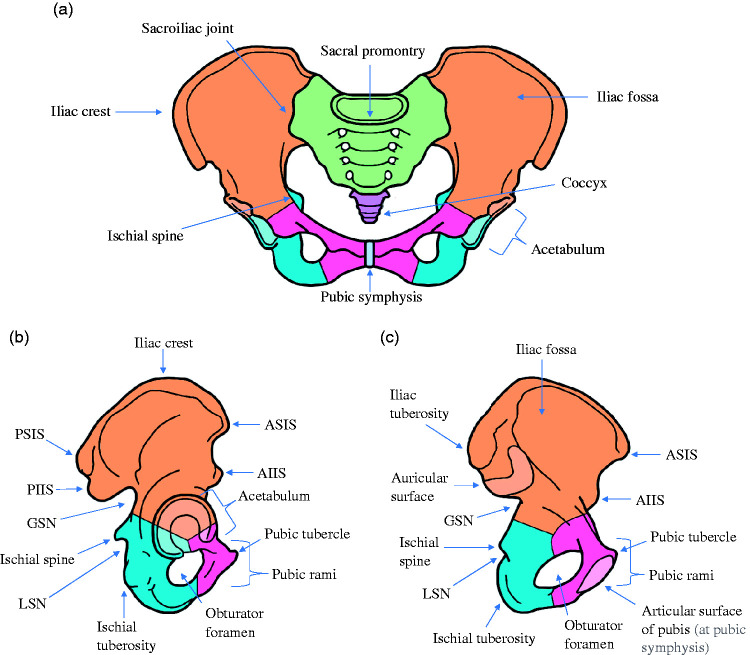
Anatomy of the pelvis. (a) Anterior view. (b) Lateral view. (c) Medial view. Orange: ileum; pink: pubis; blue: ischium; green: sacrum; purple: coccyx. ASIS: anterior superior iliac spine. AIIS: anterior inferior iliac spine; PSIS: posterior superior iliac spine; PIIS: posterior inferior iliac spine; GSN: greater sciatic notch; LSN: lesser sciatic notch

The bony pelvis consists of two hip bones, the sacrum and the coccyx (DeLancey 2008). Each hip bone is comprised of three parts: the ilium, pubis and ischium, which fuse by reproductive age (DeLancey 2008). Laterally, the hip bone creates a cup-shaped socket known as the acetabulum which articulates with the head of the femur to form the hip joint (Mahadevan 2018). The bony anatomy of the pelvis is represented in Figure 1. Strong surrounding ligamentous structures add stability to the pelvic ring. The pelvis contains the urogenital and female reproductive organs, internal iliac vessels and their branches, as well as a large variety of nerves including the lumbosacral plexus (DeLancey 2008). As such, various structures are vulnerable to injury in cases of pelvic fracture (Lee and Porter 2007).

### Preoperative considerations

#### Presentation

In cases of pelvic fracture, it is vital to ascertain the mechanism of injury as this directs the assessment and management of these patients (Langford et al 2013). Low-energy trauma should be distinguished from high-energy injury, in which significant visceral injury and haemorrhage should be anticipated (Langford et al 2013). Conscious patients may also present with pain in the pelvis, lower back, groin and hips (Lee & Porter 2007). The ability to bear weight should be noted, although being able to walk after the incident does not necessarily signify an intact pelvis (Lowth 2015).

Examination should include inspection of the skin for open wounds, swelling, contusions or degloving (known as a Morel-Lavallée lesion), and an assessment of the position and symmetry of the legs, which may be shortened and/or externally rotated (Langford et al 2013). The pelvis will commonly be unstable in high-energy fractures, although obvious deformity may not be visible (Lowth 2015). On palpation, tenderness and/or crepitus of the pubis, iliac bones, hips and sacrum may be noted (Lowth 2015). ‘Springing’ of the pelvis to identify tenderness or instability is not recommended as it does not reliably predict pelvic fractures and may cause further bleeding (Lee & Porter 2007). The hip should also be assessed for signs of acetabular fracture, such as pain on movement and instability on adduction (Lowth 2015). A peripheral vascular and neurological examination including motor function, reflexes and sensation should be performed due to the high incidence of lumbosacral plexus injuries (Chiodo 2007, Langford et al 2013).

All patients that suffer high-energy trauma must have their perineum and genitalia assessed (BOA 2016). Men should be investigated for signs of urethral injury including haematuria, scrotal haematoma or blood at the external urethral meatus (Lowth 2015). Women need to be examined for haematuria, vaginal bleeding and a palpable fracture line during bimanual examination (Lowth 2015). A rectal examination should be performed to look for rectal bleeding, a haematoma or palpable fracture, a high-riding/boggy prostate in men and the loss of anal sphincteric tone or perirectal sensation (BOA 2016, Lowth 2015).

Whilst high-energy pelvic fractures present rapidly due to the circumstances of the trauma and the patient’s clinical condition, low-energy pelvic fractures often present less acutely and more subtly (Lowth 2015). Patients are unlikely to be haemodynamically compromised and can usually walk unaided, despite the presence of pain in the pelvic region. Some avulsion fractures during sports may even go unnoticed (Lowth 2015).

#### Initial management and investigations

As with all trauma patients, initial management of patients with pelvic fractures follows the general Advanced Trauma Life Support (ATLS) principles of ABCDE: Airway, Breathing, Circulation, Disability and Exposure (Saxena et al 2014). Patients suspected of having an isolated pelvic fracture should be transferred to the nearest general hospital, whereas haemodynamically unstable or polytrauma patients should be transferred directly to a Major Trauma Centre (MTC) if possible or once resuscitated at a trauma unit (BOA 2018, NICE 2016a). MTCs are specialist centres that provide easy access to imaging, emergency operating theatres and consultant-led rehabilitation care (Moran et al 2018).

**Figure 2:**
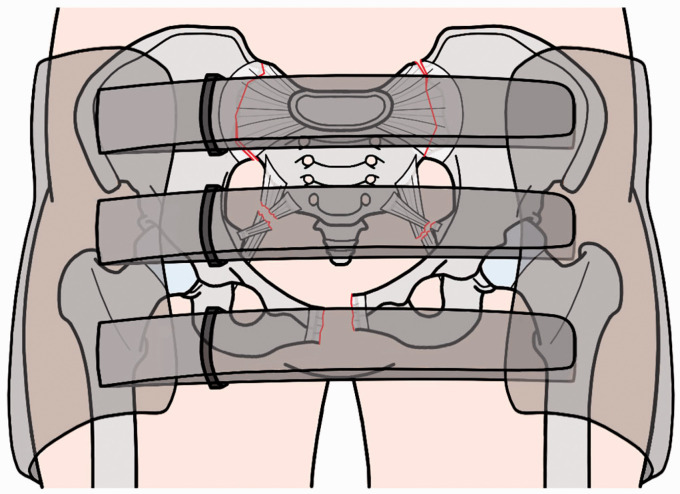
Pelvic binder. Anterior view of a fractured pelvis with a pelvic binder in place

Where there is suspicion of active bleeding, a pelvic binder must be promptly applied at the level of the greater trochanters before hospital transfer (Figure 2). This stabilises the pelvic ring, reduces bleeding from the fracture site and prevents the disruption of formed clots (BOA 2018, Lee & Porter 2007). Pain should also be regularly assessed if possible and in the context of a suspected high-energy pelvic fracture, dose-adjusted intravenous (IV) morphine is given as a first-line treatment (NICE 2016b). In cases of low-energy pelvic fractures, paracetamol is offered every six hours, with the addition of opioids if necessary (NICE 2016b). Patients are given IV tranexamic acid, ideally within one hour of injury, to treat or prevent excessive blood loss (BOA 2018). Similarly, prophylactic IV antibiotics should be given within one hour of injury for open pelvic fractures (NICE 2016a).

On admission, all patients with a suspected pelvic fracture should have the following blood tests: full blood count, coagulation tests, liver function panel, renal function tests and blood type (Graf 2018). Low-energy pelvic fracture may then be investigated using plain X-ray films, including an anteroposterior pelvic radiograph and where rami and/or sacral fractures are identified, inlet and outlet view of the pelvis (O’Connor & Cole 2014). In comparison, patients with a suspected high-energy pelvic fracture require a contrast-enhanced computer topography (CT) scan of the head, chest, abdomen and pelvis, as well as a head to toe scanogram (BOA 2018). If an active arterial pelvic bleed is identified and/or the patient is not responding to resuscitation, invasive haemorrhage control is required. In patients that do not require an emergency laparotomy for abdominal injuries, first-line treatment is interventional radiology with selective embolisation of bleeding arterial vessels (NICE 2016a). Where a laparotomy is required, this should occur with the pelvic binder in place and haemorrhage control is achieved using intraoperative pelvic packing (BOA 2018, NICE 2016a).

Pelvic binders should be removed as soon as a pelvic fracture is confirmed as mechanically stable, if the binder is failing to control the stability of the fracture, or following haemostasis (NICE 2016a). All binders should be removed within 24 hours to prevent pressure sores, but a management plan must be agreed with a pelvic surgeon beforehand in cases of mechanically unstable fractures (NICE 2016a). Polytrauma patients should have an X-ray after binder removal, even in the presence of a negative CT scan, as pelvic binders can mask significant pelvic ring ligamentous injuries (BOA 2018).

Pelvic fracture patients are also at an increased risk of urological injury (Giannoudis et al 2007). As a result, there should be one gentle attempt at urethral catherisation and where blood-stained urine is identified, a retrograde cystogram should be performed (BOA 2016). If the catheter does not pass, or passes and drains blood, it should be removed without balloon inflation and a retrograde urethrogram performed to assess for bladder or urethral injury (BOA 2016). A percutaneous suprapubic catheter can be inserted as an alternative to the urethral catheter (BOA 2016). In patients where urine leak from the bladder or urethra occurs, a closed pelvic fracture should be treated with antibiotics for 72 hours and early fracture fixation if possible (BOA 2016).

Patients with an open pelvic fracture associated with wounds to the lower abdomen, groin, buttocks, perineum, anus and rectum need urgent assessment by a colorectal or general surgeon and wound debridement (BOA 2017, 2018). Where an anal or rectal injury is suspected clinically or radiologically, a defunctioning stoma should be considered to allow for wound care of the buttocks or perineum (BOA 2018). These decisions should be made carefully as stoma formation is associated with morbidity (BOA 2018).

**Figure 3:**
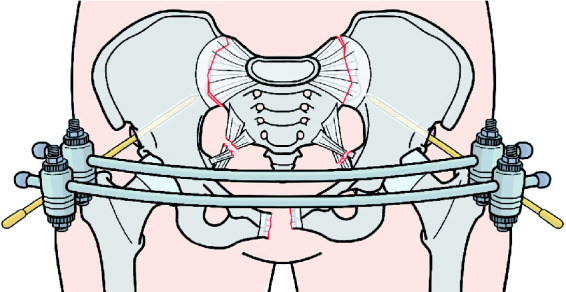
External fixation. Anterior view of a fractured pelvis treated with external fixation

Following initial management, pelvic fracture patients should undergo timely orthopaedic surgery (BOA 2018). If early definitive surgery cannot be performed, external fixation can be used to provide temporary mechanical stabilisation (Figure 3; BOA 2018). In external fixation, pins are inserted at the anterior inferior iliac spine and are directed under radiographic guidance towards the posterior ilium, just superior to the greater sciatic notch (Langford et al 2013b). Similarly, traction should be considered for patients with displaced vertical shear fractures that cannot receive early definitive surgery (BOA 2018).

#### Classification

Imaging can be used to classify pelvic fractures and can be achieved using the Academic of Orthopaedics and Orthopaedic Trauma Association, Tile, or Young-Burgess classification systems (Cheung et al 2019). The Young-Burgess classification is the most popular method and has been incorporated into the Advanced Trauma Life Support guidelines (ACOS 2004, Cheung et al 2019).

**Figure 4:**
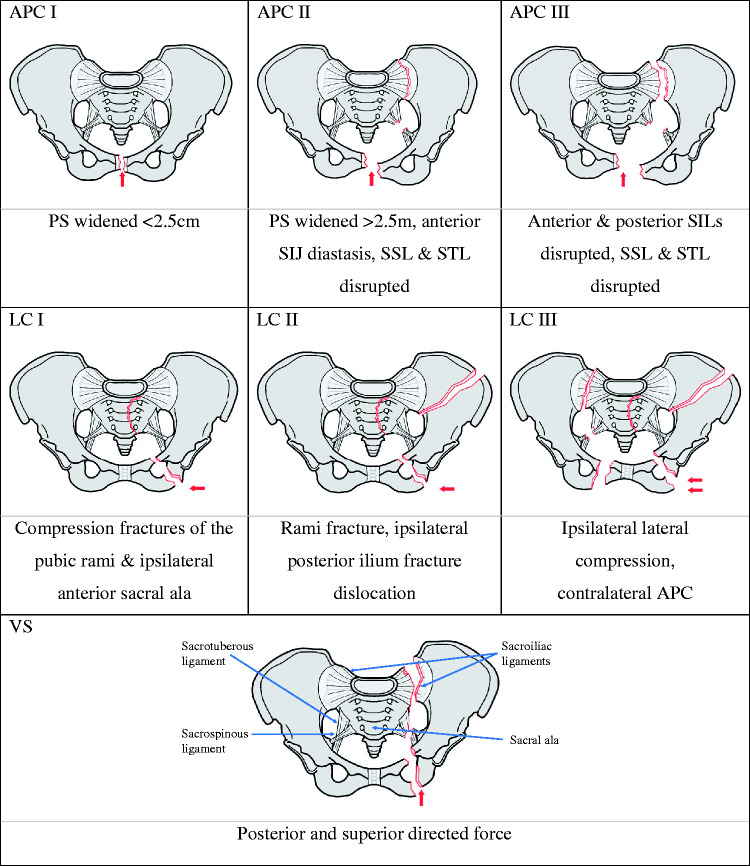
Young-Burgess classification APC I-III: anterior posterior compression fractures I-III (3%); LC I-III: lateral compression fractures I-III (89%); VS: vertical shear fracture (3%). Combined mechanism in 5%. Incidence based on Leach et al (2019). Figure based on Young et al (1986). SIJ: sacroiliac joint; SIL: sacroiliac ligament; SSL: sacrospinous ligament; ST: sacrotuberous ligament

The Young-Burgess classification (Figure 4) describes the pelvic radiographs, fracture mechanism and informs possible causes, for example, vehicle rollover or pedestrian versus auto for Lateral Compression III fractures (Weatherford 2020). It has also been reported to predict transfusion requirements, mortality and associated non-orthopaedic injuries (Manson et al 2010). The predictive value may be improved by dividing fractures into stable (APC and LC I) and unstable (all other) subtypes (Manson et al 2010). Nonetheless, it is generally accepted that patients with vertical shear fractures have the poorest outcomes; 78% present with haemorrhagic shock and 58% die (Caillot et al 2016). Given the above, it is therefore commonly used to inform treatment.

### Intraoperative considerations

**Figure 5:**
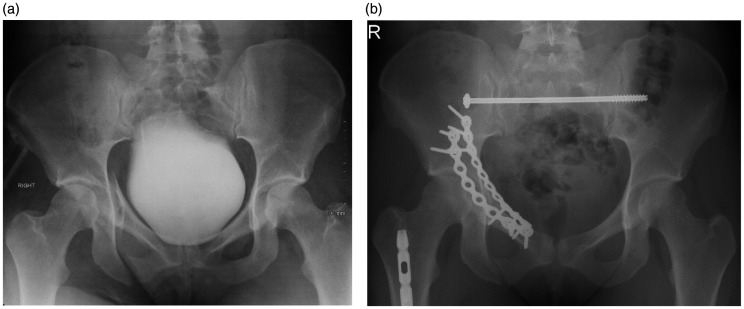
Open reduction internal fixation (ORIF). (a) Preoperative anteroposterior radiograph of a pelvic fracture with bladder contrast. (b) Postoperative radiograph of the pelvis after open reduction and internal fixation

The BOA recommends that pelvic ring reconstruction surgery should be carried out within 72 hours of the patient being in a physiologically stable state, if associated injuries allow (BOA 2018). This involves reduction of the fracture as required and surgical fixation of the pelvis (Figure 5). Reduction can be achieved percutaneously or via an open approach according to the fracture type and clinical context. Following reduction, pelvic fractures are fixed to promote stability and union, either via an anterior or posterior fixation approach, or both depending on the site of injury (Langford et al 2013b). The decision to operate and which fixation method to use can be guided by the Young-Burgess classification, stability of the injury (which may be assessed by examination under anaesthetic in some cases), patient-specific factors and the surgeon’s preference (Langford et al 2013b).

Anterior fixation can be achieved using internal fixation and/or external fixation. Internal fixation involves using an anterior intrapelvic/modified Stoppa approach through a Pfannenstiel incision to apply a multi-hole plate across the pubic symphysis (Langford et al 2013b). This approach also gives the urologist adequate exposure to address any co-existing bladder injuries (Wu et al 2015). Alternatively, anterior fixation may be achieved percutaneously in selected cases using an anterior subcutaneous internal pelvic Fixator (INFIX) (Steer et al 2019). External fixation is usually used as a temporary measure before internal fixation can be arranged; however, it may also be used as a definitive treatment for unstable APC fractures accompanied by extraperitoneal bladder rupture, or in contaminated open pelvic fractures (Langford et al 2013b).

Posterior fixation commonly involves inserting percutaneous iliosacral screws, which relies on the use of guide wires and radiographic landmarks to achieve adequate fixation and avoid key structures such as the L5 and upper sacral nerve roots, iliac vessels, sacral venous plexus and the cauda equina (Langford et al 2013b). It should be noted that a variety of factors such as obesity (leading to suboptimal fluoroscopic imaging) and sacral dysmorphism may increase the risk of screw malplacement, which has been reported in up to 11% of patients (Bulut et al 2019, Chen et al 2019). In cases where the use of iliosacral screws are thought to be unsafe, alternative techniques such as open posterior fixation via tension band plating or iliac bars may be used (Langford et al 2013b).

Due to the prevalence of associated injuries in high-energy pelvic fractures, non-orthopaedic surgery is also often indicated. This may take the form of an emergency laparotomy with pelvic packing to limit haemorrhage and address visceral injury such as an intraperitoneal bladder rupture (BOA 2016, 2018). For extraperitoneal bladder or bladder neck ruptures, the BOA recommends that primary repair should take place at the same time as pelvic ring fixation, even if the bladder injury is identified during the surgical procedure (BOA 2016). Although primary re-alignment of the urethra during fracture surgery is not recommended, primary urethral repair within 48 hours is indicated in patients with associated anorectal injury, perineal degloving, bladder neck injury, massive bladder displacement and penetrating trauma to the anterior urethra (BOA 2016).

### Postoperative considerations

Complications are commonly seen in cases of high-energy pelvic fractures, and patients often require significant surgery, which may also be a cause of morbidity (Pavelka et al 2013). This means that it can be difficult to determine whether the injury or treatment underlies each complication (Pavelka et al 2013).

Infection is a life-threatening complication that may occur in open pelvic fractures, and/or after surgical pelvic fixation. Sagi et al reported deep postoperative wound infection in 2.9% of patients requiring operative fixation, and an increased risk in those that required preoperative angio-embolisation or had a BMI greater than 30 (Sagi et al 2013). Further risk factors for infection include diabetes, prolonged operation time, prolonged ICU stay and associated genitourinary or abdominal trauma (Weatherford 2020). At-risk patients should be monitored carefully and treated with a low index of suspicion for deep pelvic infections (Sagi et al 2013). Treatment consists of high-dose systemic antibiotic therapy (Grotz et al 2005).

Pelvic fracture patients are at an increased risk of developing a venous thromboembolism (VTE), including deep vein thrombosis (DVT) and pulmonary embolism (PE), due to a prolonged period of inactivity, damage to the vascular endothelia by trauma, and surgical manipulation (Wang et al 2019). In England, the rates of DVT and PE in cases of surgically managed pelvic and acetabular fractures have been reported as 10% and 5%, respectively (Steele et al 2005). To minimise the likelihood of developing a VTE, the BOA recommends that thromboprophylaxis should be initiated according to local guidelines (BOA 2018). Steele et al recommend that anticoagulation should be given within 24 hours of injury or on achieving haemodynamic stability, as this was shown to decrease the incidence of proximal DVT (Steele et al 2005).

Urogenital injuries occur in 3.7% of pelvic fracture patients, resulting in a high prevalence of sexual and urinary dysfunction (BOA 2016, Giannoudis et al 2007). Sexual dysfunction is a known predictor of decreased quality of life and figures are as high as 43.8% in females and 52.1% in males (Harvey-Kelly et al 2014). Urethral injuries are also common, affecting between 1.6 and 25% of pelvic fracture patients, and can result in recurrent stricture formation in males with rates varying depending on the treatment method used (Barratt et al 2018). According to a study from Canada, females patients that have undergone operative pelvic fracture repair are three times more likely to require additional surgery for stress urinary incontinence, compared to the general population (Welk et al 2015). Currently, the BOA recommends that an information sheet be given to all patients regarding sexual and urinary dysfunction (BOA 2016).

In the long-term, 64% of pelvic ring fracture patients complain of chronic post-traumatic pelvic pain, even 52 months after the injury (Gerbershagen et al 2010). Anxiety and depression correlate moderately to strongly with the chronicity stage of this pain (Gerbershagen et al 2010). These patients tend to have a lower quality of life compared to the general population, including a long-term decrease in physical functioning, problems with activities of daily living and adverse psychological health (Banierink et al 2019). Increasing age, complex trauma and surgical management are all predictors of a poor quality of life amongst pelvic fracture patients (Holstein et al 2013). All patients should attend a follow-up appointment in a specialist pelvic trauma unit or rehabilitation clinic to facilitate the optimal management of pain, physical, psychological and urological disabilities (BOA 2018).

## Conclusion

Pelvic fractures are serious injuries with significant morbidity and mortality, especially in the perioperative period. As a result, early recognition, work-up and appropriate perioperative management are required to optimise the care that these patients receive.

## Key phrases

1. Pelvic fractures are commonly associated with other injuries and should be managed by a multidisciplinary team.
2. Patients with pelvic fracture require thorough assessment and timely surgery.
3. Pelvic fractures can be classified according to their radiological appearance.
4. There are multiple physical and psychosocial complications of pelvic fractures.
